# Retrospective analysis on the efficacy of epidural labor analgesia on early breast feeding after vaginal delivery

**DOI:** 10.1186/s12871-023-02373-w

**Published:** 2023-12-14

**Authors:** Xudong Hu, Dongqin Xiong, Meifang Luo, Chen Ling, Xingqing Liu, Kai Yang, Xianjie Wen

**Affiliations:** 1 Department of Anesthesiology, The Second People’s Hospital of Foshan, Weiguo Road NO78, Destrict of Chancheng, Foshan City, Guangdong Province China 528000; 2 Department of Obstetrics, The Second People’s Hospital of Foshan, Weiguo Road NO78, Destrict of Chancheng, Foshan City, Guangdong Province China 528000

**Keywords:** Epidural labor analgesia, Vaginal delivery, Efficacy, Breastfeeding, LATCH score, Retrospective analysis

## Abstract

**Background:**

Breastfeeding is essential for infants and mothers. Epidural labor analgesia is used frequently to alleviate pain during vaginal delivery. Studies have found that epidural labor analgesia potentially have negative effects on postpartum breastfeeding. However, the efficacy of epidural labor analgesia on early breastfeeding after vaginal delivery is unclear. Therefore, a retrospective analysis was performed to illuminate the efficacy of epidural labor analgesia on postpartum breast feeding.

**Methods:**

A total of 392 women who received vaginal delivery in the Second People’s Hospital of Foshan from July 2022 to June 2023 were selected for this study, and all women received epidural labor analgesia and were divided into three groups according to the efficacy of labor analgesia. There were three groups: parturients with VAS scores < 3 were divided into Group E (*n* = 192), parturients with VAS scores 4–6 were divided into Group M (*n* = 127), and parturients with VAS scores > 7 were divided into Group P (*n* = 73). The labor process, lactation initiation time, and incidence of delayed onset of lactation were analyzed. The lactation volume and time and LATCH score at 24, 48 and 72 h after vaginal delivery were also analyzed.

**Results:**

There was no significant difference in labor process times among the three groups (*P* > 0.05). The cases of prolactin use in Group M were less than those in Group E and Group P, with a significant difference (all *P* < 0.05). There was no significant difference in cases of prolactin use between Group E and Group P (*P* > 0.05). The lactation initiation time in Group M was significantly shorter than those in Group E and Group *P* (all *P>*0.05). There was no significant difference in lactation initiation time after vaginal delivery between Group E and Group P (*P*>0.05). The incidence of delayed onset of lactation in Group M was significantly lower those that in Group E and Group P (all *P* < 0.05). There was no statistically significant difference in the incidence of delayed onset of lactation between Group E and Group P (*P* > 0.05). The lactation volumes at 24, 48 and 72 h after vaginal delivery in Group M were significantly higher than those in Group E and Group P (all *P* < 0.05). There was no significant difference in lactation volume at 24, 48 and 72 h after vaginal delivery between Group E and Group P (*P* > 0.05). The lactation times at 24, 48 and 72 h after vaginal delivery in Group M were significantly higher than those in Group E and Group P (all *P* < 0.05). There was no significant difference in lactation times at 24, 48 and 72 h after vaginal delivery between Group E and Group P (*P* > 0.05). There was no significant difference in LATCH scores at 24, 48 and 72 h after vaginal delivery among the three groups (all *P* > 0.05).

**Conclusions:**

Compared with labor analgesia with excellent and poor analgesia efficacy, labor analgesia with moderate analgesia efficacy has fewer cases of prolactin use, more lactation volume and time, a shorter lactation initiation time, a lower incidence of delayed onset of lactation and no effect on the LATCH score of breastfeeding.

**Supplementary Information:**

The online version contains supplementary material available at 10.1186/s12871-023-02373-w.

## Background

The World Health Organization (WHO) and the United Nations Children’s Fund (UNICEF) recommend that exclusive breast feeding for infants must last for at least 6 months and continue breast feeding until children are 2 years or older to optimize the growth, development, and health of the child [1]. According to a survey report released recently by the China Development Research Foundation, the rate of exclusive breast feeding within 6 months in China is generally low (only 29.2%), which is far below the goal of 50% by 2020 set by the “China Child Development Program“ [2]. Breast feeding has many health benefits for infants and later children and for mothers, and how to increase the incidence of breast feeding is very important [3]. Studies have found that there are many factors that affect breast feeding, including complications, age, parity of delivery woman, mode of vaginal delivery, labor analgesia, maternal culture, breast feeding education, family income, family support, and newborn conditions. Pain during delivery is an important factor [4].

 Delivery is very painful in both primiparas and multiparas, and the parturient suffers from more severe pain during delivery [5]. Pain during vaginal delivery includes visceral and somatic pain, which results in an emotionally unpleasant situation for the parturient and has harmful effects on both the mother and baby. Pain can stimulate the release of catecholamines, which can cause uterine vasoconstriction. Pain can also cause maternal hyperventilation, leading to hypocapnia, which further constricts uterine blood vessels, reduces the driving force of the parturient during uterine contraction and leads to a change in the parturients oxygen dissociation curve, which shifts to the left and then impairs fetal oxygen supply and may lead to fetal hypoxemia and metabolic acidosis. Painful labor can also lead to maternal birth canal injury and fetal injury [6]. Therefore, labor analgesia should not only reduce the pain of the mother but also make the labor process safer for both the mother and baby and should not affect breast feeding. The labor epidural analgesia is fulfilment by the epidural injections of a mixture of a low concentration of local anesthetic (bupivacaine or ropivacaine) and a small dose of a fat-soluble opioid (sufentanil or fentanyl). Recent literature found that other adjuvants, such as dexmedetomidine or clonidine, have proven their efficacy both for epidural analgesia [7, 8] and for spinal anesthesia for C-Sect. [9].

In epidural labor analgesia, due to the use of local anesthetics, the T8-S spinal nerve root of the puerperal was blocked, and the pain during delivery was eliminated or mitigated [10]. A large number of studies have reported that epidural labor analgesia can effectively relieve the pain of parturients during delivery with few adverse reactions. Epidural analgesia is currently the gold standard for labor analgesia [10]. It has been reported that labor analgesia can increase the secretion of prolactin and milk production [11, 12]. Some scholars believe that epidural labor analgesia does not affect prolactin levels or lactation in mothers [13]. La Camera et al. found that the effects of epidural analgesia on stress markers are beneficial to the newborn and do not significantly influence the newborn’s well-being during labor and development [14].

Callahan et al. found that epidural labor analgesia is not consistently associated with any significant adverse outcomes, including transient maternal hypotension, fever in parturients, decreased Apgar scores in newborns, or poor acid‒base neutralization. The above results confirmed the safety of epidural labor analgesia for newborns and parturients. Studies have found that epidural labor analgesia does not affect the parturient’s lactation initiation time [15, 16]. However, some studies found that epidural labor analgesia can prolong the lactation initiation time [17, 18]. Moreover, most current studies on epidural labor analgesia use parturients without labor analgesia as the control group, and the results cannot reflect the different analgesic efficacy of breast feeding. Due to the inconsistent effect of epidural labor analgesia, the pain perception of the parturients is also inconsistent, which may affect breast feeding.

This study retrospectively analyzed the effects of different analgesic efficacy on the lactation initiation time, the incidence of delayed onset of lactation and lactation volume and times and the LATCH score of the parturient’s breast feeding after vaginal delivery.

## Methods and information

### Case selection and grouping

**Fig. 1:**
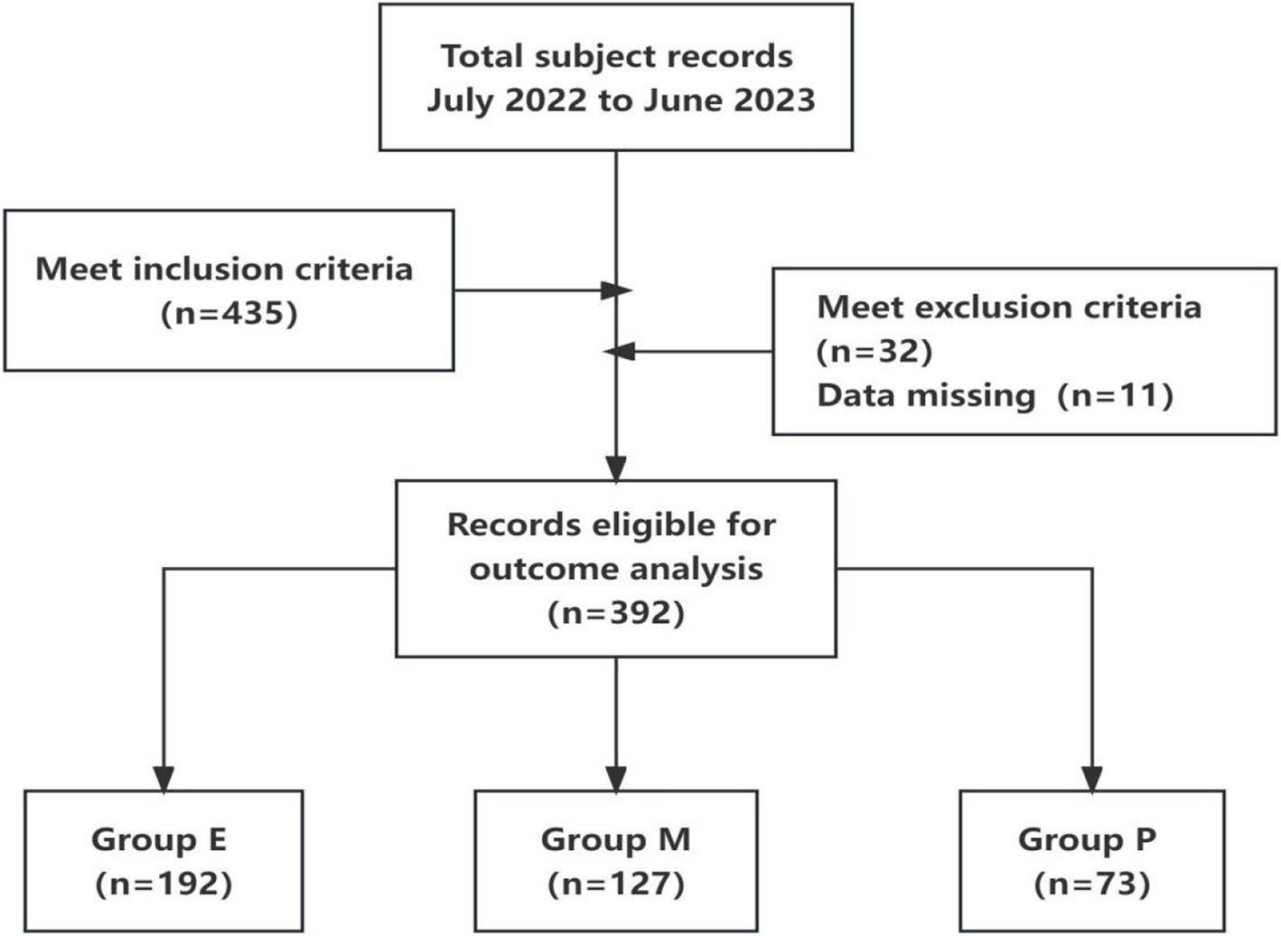
Flow chart describing the process of inclusion and exclusion of cases for the purpose of participation eligibility

A total of 495 parturients of full-term gestation who received vaginal delivery in the Department of Obstetrics of the Second People’s Hospital of Foshan from July 2022 to June 2023 were selected. There were 435 parturients meeting the inclusion criteria in the 495 parturients. There were 32 parturients meeting the exclusion criteria and 11 parturients with missing data in the 435 parturients .So a total of 392 parturients with records eligible for outcome analysis were divided into three groups: Group E (VAS < 3 points), Group M (VAS 4–6 points), and Group P (VAS > 7 points). A total of 192 cases were in Group E, 127 cases in Group M and 73 cases in Group P according to the VAS score during vaginal delivery. The study was approved by the Ethics Committee of the Second People’s Hospital of Foshan with document number 2023-0066, and written informed consent was signed by the parturient or her family members.The process of inclusion and exclusion of cases for the purpose of participation eligibility in the present study is described in Fig. 1.

The inclusion criteria were as follows: (1) 25-35-year-old parturient with single full-term labor; (2) ASA classification I-II; (3) parturient who met the indications for vaginal delivery; (4) parturient who had no contraindications for epidural analgesia; (5) parturient without heart, kidney, or liver disease; and (6) parturient without combined severe pathological obstetrical conditions such as severe preeclampsia and pregnancy-induced hypertension syndrome.

Exclusion criteria: (1) Abnormal labor process, difficult vaginal delivery and conversion to cesarean section; (2) Bleeding > 700 ml in 24 h after vaginal delivery; (3) Low weight or macrosomia of newborn (newborn weight < 2500 g or > 4000 g); (4) Aparg score of the newborn is < 8 points; (5) The newborn has indications for artificial feeding; (6) Mother-infant separation; (7) Abnormal fetal position.

### Protocol of labor analgesia

All parturients were undergoing epidural labor analgesia for vaginal delivery. When the cervix was dilated to 2 cm, epidural puncture was performed at the L2-3 intervertebral vertebra, and an epidural catheter was inserted into the epidural space. A 10 ml mixture of 0.1% ropivacaine (including sufentanil 5 µg) was injected into the epidural space through the catheter, and the epidural catheter was connected to the self-controlled electronic analgesia pump. The parameters of the analgesic pump were set to the background with infusion at a rate of 7–8 mL/h, a PCA bolus volume of 5 ml and a locking time of 15 min. If the parturient feels that the analgesic effect is unsatisfactory, the parturient or midwife can press the automatic control switch. After vaginal delivery, the analgesic pump infusion was stopped.

The dose of 400 mg ibuprofen was administrated orally and can be repeated every four to six hours ,if necessary with a maximum daily dose of 2400 mg.

The formula of analgesic solution was sufentanil citrate 50 µg (Jiangsu Huahua Pharmaceutical Co., Ltd., National Drug Approval: H2020365336, specification: 10 mL: 50 µg, product batch number: SZ221202) and 1% ropivacaine hydrochloride 10 ml (AstraZeneca AB, import registration certificate number: H20140763, specification: 10 ml: 100 mg, product batch number: NBMB) added to 80 ml of normal saline.

## Observation

### General data of the parturient

The general characteristics of the parturient, such as age, height, weight, gestational week, number of pregnancies, number of laborers, education during pregnancy, education of the parturient and family income, were all recorded.

### Delivery pain score

VAS was used to score the pain during vaginal delivery, and the VAS score ranged from 0 to 10, with greater scores correlating severe the pain. The VAS was evaluated every hour after the onset of labor analgesia, the lowest VAS value throughout the delivery was recorded for grouping, and remedial analgesia was not implemented for parturients with unsatisfactory analgesia.

### Maternal vaginal delivery

The time of the first, second, third and the total stages of labor, the method of membrane rupture, the use of cervical balloon, the use of oxytocin, the manual fetal head rotation, the case with vaginal lateral cut, the fetal position, the neonatal sex, and the neonatal birth weight, were recorded.

### Maternal lactation times

The lactation initiation time refers to the time when the parturient feels the first breast swell or the milk can flow slowly when squeezed by fingers on the breast. The delayed onset of lactation was diagnosed if her lactation initiation time was longer than 72 h.

### Maternal lactation volume after vaginal delivery

First the newborn was weighed, the mother breast fed the newborn, and the newborn was weighed again after breast feeding. The lactation volume = weight after breast feeding - weight before breast feeding. Maternal lactation volume was recorded at 24, 48 and 72 h after vaginal delivery.

### Maternal lactation times after vaginal delivery

Mothers were asked to breast feed their newborns once they left the vaginal delivery room, and maternal lactation times at 24 h, 48 and 72 h after vaginal delivery were recorded.

### LATCH score after vaginal delivery

Rapheal et al. [19] The LATCH score was judged at 24, 48 and 72 h after vaginal delivery. The LATCH score is a key component of breast feeding, including 5 items, and each item was assigned a numerical score of 0, 1, or 2. The result of adding the value of 5 items is the LATCH score.

### Statistical analysis

SPSS 23.0 software was used for statistical analysis. Measurement data are expressed as the mean ± standard deviation (‾ χ ± S). The comparison of measurement data was used for one-way ANOVA to compare differences between the three groups. Two-way ANOVA was used to compare differences between the groups and the time points. If the difference was statistically significant, the post hoc LSD test was performed. The data distribution curve followed either a normal distribution or an approximately normal distribution in our study, which was judged by quantile‒quantile blot. The enumeration data were expressed as constituent ratios and rates, and the chi-square test or Fisher’s exact probability calculation method was used for comparison among the three groups. Rank data were statistically analyzed by Mann‒Whitney tests. *P* < 0.05 was considered statistically significant.

## Results

### General data

There was no statistically significant difference in maternal age, height, weight, gestational week, number of pregnancies, number of laborers, pregnancy education, maternal education or family income (*P* > 0.05). As shown in Table 1.

**Table 1:** Comparison of the general status (χ ± S)

Grouping	Group E (*n* = 192)	Group M (*n* = 127)	Group p (*n* = 73)	F(χ2)value	*P* value
Age (year)	28.59 ± 4.36	28.27 ± 4.69	28.74 ± 4.38	0.312	0.732
Height (cm)	163.22 ± 13.55	162.23 ± 13.85	158.97 ± 8.82	1.882	0.154
Weight (kg)	67.69 ± 9.79	66.59 ± 10.16	68.54 ± 8.67	0.997	0.370
Gestation ( weeks )	39.47 ± 1.04	39.46 ± 0.97	39.41 ± 1.08	0.106	0.899
Gravidity	1.80 ± 0.93	1.82 ± 1.10	1.73 ± 1.04	0.203	0.816
Parity	1.43 ± 0.58	1.42 ± 0.67	1.33 ± 0.53	0.797	0.451
Pregnancy education (Y/N)	133/59	92/35	43/20	1.571	0.456
Maternal education				1.035	0.904
Below high school	40	27	12		
Junior college and undergraduate	129	84	53		
Postgraduate	23	16	8		
Family income per year				4.485	0.344
> 20,000yuan (RMB)	39	24	16		
10,000–20,000 yuan (EMB	91	73	33		
< 10,000 yuan (RMB)	62	30	24		

### Vaginal delivery conditions

There was no significant difference in the mode of rupture of the membranes, the cases of cervical balloon use, the cases of manual fetal head rotations, the cases of vaginal lateral cut, the fetal position, the newborn weight after birth.There was a statistically significant difference in the incidence of oxytocin use during vaginal delivery among the three groups (*P* = 0.041, *P* < 0.05). The incidence of oxytocin use during vaginal delivery in Group E was significantly higher than that in Group M and Group P (*P* = 0.027, 0.030, all *P* < 0.05). There was no statistically significant difference in the incidence of oxytocin use during vaginal delivery in Group M and Group P (*P* = 0.658, *P* > 0.05). There was a significant difference in the VAS scores among the three groups during vaginal delivery (*P* = 0.000, *P* < 0.05). The VAS score during vaginal delivery in Group E was significantly lower than those in Group M and Group P (*P* = 0.000, *P* < 0.05). The VAS score during vaginal delivery in Group M was significantly lower than that in the Poor group (*P* = 0.000, *P* < 0.05). As shown in Table 2.

**Table 2:** Comparison of maternal delivery (χ ± S)

Grouping	Group E (*n* = 192)	Group M (*n* = 127)	Group P (*n* = 73)	F(χ^2^)value	*P* value
First stage of labour (min)	438.05 ± 211.13	422.58 ± 191.57	438.77 ± 204.43	0.254	0.776
Second stage of labour (min)	74.05 ± 59.69	92.31 ± 166.24	68.38 ± 47.35	1.572	0.209
Third stage of labour (min)	11.07 ± 7.18	11.15 ± 6.68	10.124.33	0.666	0.514
Total stage of labour (min)	527.70 ± 237.45	547.97 ± 280.66	528.71 ± 228.83	0.271	0.763
Debranching mode Artificial/Early stage	97/49/46	67/34/26	31/28/14	5.003	0.287
Cervical balloon using (cases)	35	21	6	4.053	0.132
Oxytocin use (cases)	81^#^	38	33^#^	6.41	0.041
Fetal head change with hand (cases)	5	3	3	0.074	0.964
Vaginal lateral incision (cases)	51	26	15	2.005	0.367
Fetal position				0.074	0.964
LOA	155		60		
ROA	34	21	13		
Others	3	3	0		
Neonatal birth weight (g)	3.15 ± 0.43	3.18 ± 0.46	3.15 ± 0.44	0.178	0.836
VAS during labour	1.66 ± 0.93	4.93 ± 0.78	7.81 ± 0.78	148.29	0.000

^#^Compare with Group M, *P*<0.05

#### Lactation initiation time and the incidence of delayed onset of lactation

**Fig. 2:**
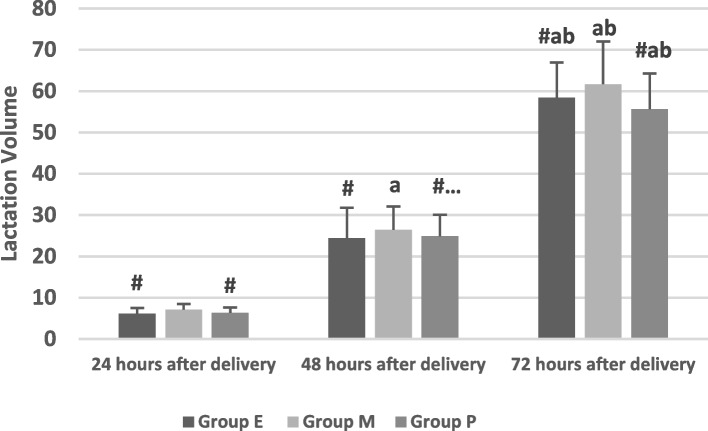
Comparison of the lactation initiation time and the incidence of delayed onset lactation of the three groups

There was a statistically significant difference in the initiation time of lactation after vaginal delivery among the three groups (F = 4.604, *P* = 0.011, *P* < 0.05), and the lactation initiation time in Group M was shorter than that in Group E (*P* = 0.006, *P* < 0.05). There was no significant difference in the lactation initiation time between Group E and Group P (*P* = 0.825, *P* > 0.05). There were 24 cases (12.50%), 6 cases (4.72%), and 10 cases (13.7%) of delayed onset of lactation among the three groups, respectively, with significant differences (χ^2^ = 6.239, *P* = 0.044, *P* < 0.05). The incidence of delayed onset of lactation in Group M was less than that in Group E, with a significant difference (*P* = 0.020, P < 0.05). There was no significant difference in the delayed onset of lactation between Group M and Group P (*P* = 0.051, *P* > 0.05). There was no significant difference in the delayed onset of lactation between Group E and Group P (*P* = 0.794, *P* > 0.05). As shown in Table 3 , Fig.  2.

**Table 3:** Comparison of breast-feeding status (χ± S)

Grouping	Group E (*n* = 192)	Group M (*n* = 127)	Group P (*n* = 73)	F(χ^2^)value	*P* value
Lactation initiation time	53.75 ± 10.36^#^	50.56 ± 9.64	54.05 ± 8.79^#^	4.604	0.011
Incidence of delayed onset of lactation	24 (12.50%)^#^	6 (4.72%)	13 (13.70%)^#^	6.678	0.036
Lactation volume					
24 h	6.16 ± 1.35^#^	7.11 ± 1.40	6.33 ± 1.34^#^	19.177	0.00
48 h	24.42 ± 7.35^#,a^	26.42 ± 5.64^a^	24.86 ± 5.25^a,b^	3.737	0.025
72 h	58.41 ± 8.51^#,a,b^	61.67 ± 10.36^b^	55.68 ± 8.63^#,a,b^	10.538	0.000
Lactation times					
24 h	6.94 ± 1.07^#^	7.23 ± 1.42	6.85 ± 1.41	2.836	0.060
48 h	7.02 ± 0.95^#^	7.59 ± 1.50^a^	7.11 ± 1.16^#,a^	9.271	0.000
72 h	7.33 ± 1.13^a,b^	7.20 ± 1.16^b^	6.77 ± 1.35^#^	6.077	0.003
LATCH scores					
24 h	7.58 ± 1.54	7.58 ± 1.25	7.10 ± 1.29	2.755	0.065
48 h	7.52 ± 1.28	7.69 ± 1.03	7.70 ± 1.27	1.036	0.356
72 h	7.50 ± 1.63	7.62 ± 1.54	7.85 ± 1.50	1.396	0.249

^#^Compared with Group M, *P* <0.05

^a^Compared with at 24 h, *P* <0.05

^b^Compared with at 48 h, *P* <0.05

#### Lactation volume

There was no significant difference in lactation volume between Group E and Group P at 72 h after vaginal delivery (*P* = 0.176, *P* > 0.05). The lactation volume at 72 h after vaginal delivery in Group M was greater than that in Group E and Group P (*P* = 0.000, 0.000, all *P* < 0.05). There was a statistically significant difference in the lactation volume at 24, 48 and 72 h after vaginal delivery in all groups. (*P* = 0.000, 0.000, 0.000, all *P* < 0.05). The lactation volume at 24 h after vaginal delivery was significantly less than that at 48 and 72 h (*P* = 0.000, *P* < 0.05). The lactation volume at 24 h and at 48 h after vaginal delivery was significantly less than that at 72 h among the three groups (*P* = 0.000, *P* < 0.05).

**Fig. 3:**
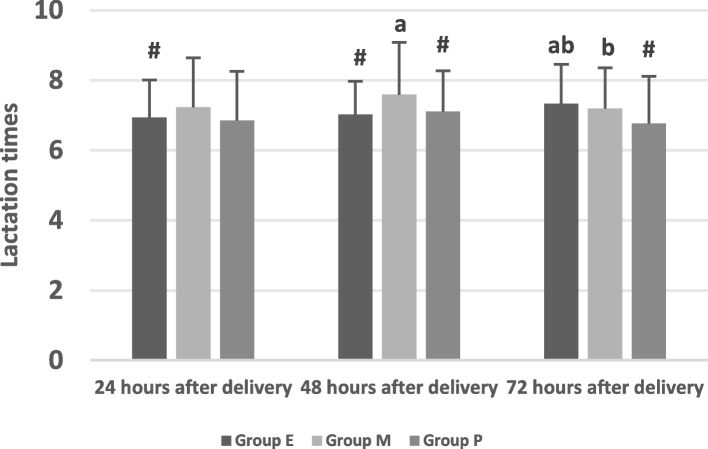
Comparison of the lactation volume of the three groups

 There was a significant difference in lactation volume at 24 h after vaginal delivery among the three groups (F = 19.177, *P* = 0.000, *P* < 0.05). The lactation volume in Group M was significantly higher than those in Group E and Group P. (*P* = 0.00, 0.00, all *P* > 0.05). There was no significant difference in lactation volume at 24 h after vaginal delivery between Group E and Group P (*P* = 0.373, *P* > 0.05). There was a significant difference in lactation volume at 48 h after vaginal delivery among the three groups (F = 9.271, *P* = 0.000, *P* < 0.05). The lactation volume at 48 h after vaginal delivery in Group M was significantly higher than that in Group E. (*P* = 0.007, *P* < 0.05). There was no significant difference in lactation volume at 48 h after vaginal delivery between Group E and Group P (*P* = 0.616, *P* > 0.05). There was a significant difference in the lactation volume at 72 h after vaginal delivery among the three groups (F = 10.538, *P* = 0.000, *P* < 0.05). The lactation volume at 72 h after vaginal delivery in Group M was significantly higher than that in Group E and Group P (*P* = 0.002,00.000, all *P* < 0.05). There was a significant difference in lactation volume at 72 h after vaginal delivery between Group E and Group P (*P* = 0.032, *P* < 0.05). As shown in Table 3, Fig. 3.

### Lactation times

There was a significant difference in the lactation times between Group E and Group P (*P* = 0.052, *P* > 0.05). The lactation times 72 h after vaginal delivery in Group M were significantly greater than those in Group E and Group P (*P* = 0.002, 0.000, all *P* < 0.05). The lactation times at 24 h after vaginal delivery among the three groups were significantly lower than those at 48 h after vaginal delivery (*P* = 0.018, *P* < 0.05). There was no significant difference in lactation time between 48 and 72 h (*P* = 0.680, *P* > 0.05). There was no significant difference in lactation time between 24 and 72 h (*P* = 0.052, *P* > 0.05).

**Fig. 4:**
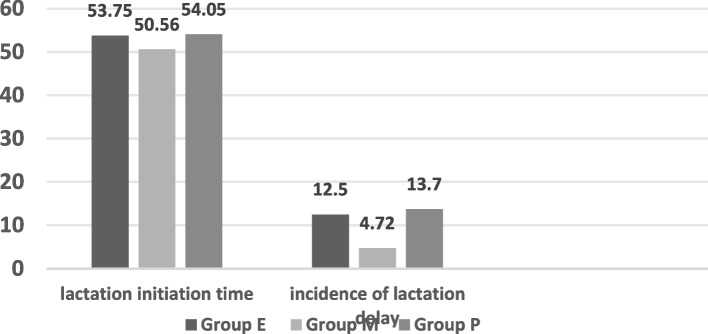
Comparison of the lactation times of the three groups

 There was a statistically significant difference in the lactation times at 24 h after vaginal delivery among the three groups (F = 12.839, *P* = 0.060, *P* > 0.05). The lactation times at 24 h after vaginal delivery in Group M were significantly greater than those in Group E and Group P (*P* = 0.000, 0.040, all *P* < 0.05). There was no significant difference in lactation times at 24 h after vaginal delivery between Group E and Group P (*P* = 0.610, *P* > 0.05). There was a statistically significant difference in the lactation times at 48 h after vaginal delivery among the three groups (F = 3.737, *P* = 0.025, *P* < 0.05). The lactation times at 48 h after vaginal delivery in Group M were significantly greater than those in Group E and Group P (*P* = 0.000, 0.006, *P* < 0.05). There was no significant difference in lactation time at 48 h between Group E and Group P (*P* = 0.567, *P* > 0.05). There was no significant difference in lactation time at 72 h after vaginal delivery among the three groups (F = 6.07, *P* = 0.003, *P* < 0.05). The lactation times at 72 h after vaginal delivery in Group M were significantly greater than those in Group P (*P* = 0.014, *P* < 0.05). The lactation times at 72 h after vaginal delivery in Group E were significantly greater than those in Group P (*P* = 0.001, *P* < 0.05). As shown in Table 3, Fig. 4.

**Fig. 5:**
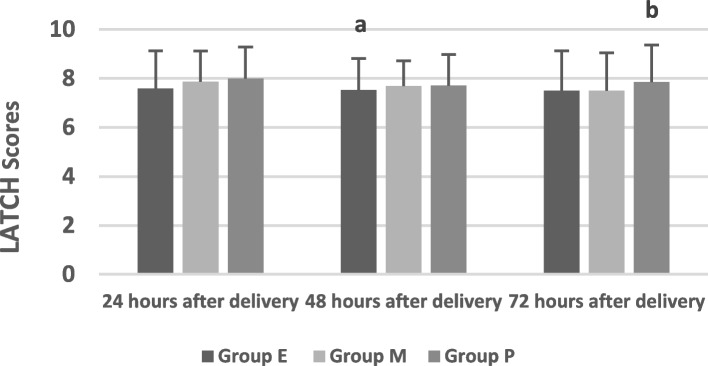
Comparison of the LATCH score of the three groups. # Compared with Group M; **a** Compared with at 24 hour, *P *<0.05; **b** Compared with at 48 hour, *P *<0.05

 There was a significant difference in LATCH score after vaginal delivery between 24 and 72 h (*P* = 0.014, *P* < 0.05). There was no significant difference in the LATCH score among the three groups at 24, 48 and 72 h after vaginal delivery (F = 1.396; *P* = 0.249; F = 1.036, *P* = 0.356; F = 2.755, *P* = 0.065, all *P* > 0.05). As shown in Table 3, Fig. 5.

## Discussion

The delivery is a natural physiological process of human reproduction, However, it can cause severe pain and unpleasant feelings for the mother. If fetal abnormalities such as fetal big head and breech presentation occur, they can cause birth canal damage and difficulty vaginal delivery. However, once the process of vaginal delivery is smooth, there are fewer complications, early sucking and fast lactation [6]. Pain during delivery may inhibit uterine contraction by stimulating the sympathetic nervous system, resulting in delayed lactation in some parturients. Studies have shown that pain during natural delivery can increase the release of β-endorphins in the body. β-Endorphins induce self-regulation of pain and promote the release of prolactin and oxytocin; the latter is beneficial to vaginal delivery and breastfeeding [20]. Moreover, in natural delivery, the fetal head may press on the birth canal and simulate the hypothalamusand pituitary through the dorsal root of the spinal nerve in the lumbosacral region, promoting the release of oxytocin and prolactin, which is conducive to contraction of the uterus and breast feeding after delivery.

Studies have found that severe pain can inhibit prolactin and oxytocin secretion via the sympathetic nervous system which secret the adrenal medullary, adrenal cortex hormones and other hormones and the analgesia, including epidural analgesia, can inhibite the excitation of the sympathetic nervous system and maintain the level of prolactin and oxytocin. However, studies have found that serum prolactin levels have increased in patients with headache, especially migraineand and surgical trauma can activate hypothalamus and pituitary. and cause the increased serum levels of prolactin and oxytocin for regulating nociceptive responses [21, 22]. Therefore the pain is a double-edged sword [23]. We speculate that different degree of pain can result in different degree of prolactin and oxytocin secretion.

At present, epidural labor analgesia is the gold standard of labor analgesia, which can effectively reduce pain during labor and reduce the probability of cesarean section, but it can delay the first stage of labor by 30 min and the second stage of labor by 15 min and increase exogenous oxytocin application and the probability of instrumental assisted vaginal delivery [24]. The reason is that epidural labor analgesia inhibits uterine contraction pain and inhibits the stimulation of the fetal head to the birth canal, thereby inhibiting the release of endogenous oxytocin from the hypothalamus. However, our study found that there was no significant difference in the first stage of labor, the second stage of labor, the third stage of labor and the total stage of labor in the three groups with different analgesic efficacies. The incidence of received exogenous oxytocin in Group M was less than those in Group E and Group P. This finding suggested that labor analgesia efficacy had no significant impact on the labor process but had an impact on the incidence of exogenous oxytocin use. We found that when the efficacy of epidural labor analgesia was better, the use of exogenous oxytocin increased. The reason may be that the better efficacy of epidural labor analgesia may decrease the secretion of endogenous oxytocin through the abovementioned mechanism. At the same time, the poor efficacy of epidural labor analgesia may also increase the use of exogenous oxytocin. This may be caused by the inhibition of the secretion of endogenous oxytocin, which was induced by the excitation of the sympathetic nervous system, which was caused by severe pain. Studies have found that epidural labor analgesia inhibits the release of endogenous oxytocin. Since oxytocin stimulates uterine contraction during labor, the lower level of endogenous oxytocin may be one of the reasons for the increased use of exogenous oxytocin [25]. Labor epidural analgesia interferes with the release of endogenous oxytocin and prolactin, resulting in decreased levels of endogenous oxytocin in parturients receiving epidural labor analgesia, which may play a role in delayed breastfeeding after vaginal delivery. Studies have confirmed that plasma levels of oxytocin are reduced during labor in parturients receiving labor analgesia [26].

Because epidural labor analgesia can block the transmission of the signal of the maternal sacral nerve and lower lumbar nerve root, it may inhibit the stimulation of the fetal head to the birth canal and cause decreased secretion of prolactin in the hypothalamus. Prolactin is a key factor in maternal lactation after delivery, and endogenous oxytocin and prolactin act synergistically to promote maternal lactation [27]. There are paradoxical results regarding the efficacy of labor epidural analgesia on breastfeeding, including promoting breastfeeding, having no effect on breastfeeding, and negatively affecting breastfeeding [10–16, 28]. Most of the studies used blank controls, which could not reflect the effect of different analgesic efficacy on breastfeeding. All parturients who received epidural labor analgesia into three groups according to pain during labor and the efficacy of epidural labor analgesia on breast feeding were studied in our study. The results suggested that different labor analgesic efficacies may have an impact on maternal breastfeeding. Studies have shown that lactation at 2 h after vaginal delivery is related to breastfeeding at 6 months after vaginal delivery [29]. After vaginal delivery and placental peeling from the uterus, the prolactin in the maternal body begins to change, and lactation begins. Lactation reaches peaks at 48–72 h after vaginal delivery, which is the second stage of lactation. The reproductive hormone oxytocin also plays an important role during lactation, as oxytocin is necessary for the milk ejection reflex and the secretion of milk from the mammary gland [30]. The use of epidural analgesia during labor can reduce maternal plasma oxytocin and prolactin levels [31], and severe pain can also reduce maternal plasma oxytocin and prolactin levels. Therefore, the efficacy of epidural labor analgesia may influence the lactation of delivery parturients, such as lactation initiation time, lactation volume and lactation time.

Studies have found that delayed onset of lactation after vaginal delivery is associated with cessation of breastfeeding for up to 4 weeks; therefore, it is important to shorten the time to breast milk onset. We observed the lactation initiation time within 72 h after vaginal delivery in the three groups and found that the lactation initiation time in Group M was the shortest among the three groups and that the lactation initiation time in Group P was the same as that in Group E. The results suggested that the better efficacy of epidural analgesia was associated with prolonged lactation initiation time. However, poor efficacy was also associated with prolonged lactation initiation time. The moderate efficacy of epidural analgesia had the shortest lactation initiation time. Studies have found that parturients who receive labor epidural analgesia have a prolonged lactation initiation time compared with women who do not receive epidural analgesia [17, 18]. The diagnosis of delayed onset of lactation is generally diagnosed when the onset time of lactation is more than 72 h after vaginal delivery [2, 18]. We found that the incidence of delayed onset of lactation was different in the three groups and that there were 24 cases (12.50%), 6 cases (72%) and 10 cases (13.70%) of delayed onset of lactation after vaginal delivery in Group E, Group M and Group P, respectively, and the incidence of delayed onset of lactation in Group M was less than that in Group E and Group P. It is suggested that although perfect analgesia can significantly reduce pain during delivery, it can also lead to prolonged lactation initiation time and increase the incidence of delayed onset of lactation after vaginal delivery [2]. Studies have found that labor epidural analgesia could delay lactation initiation time compared with no epidural analgesia [2, 18]. Our study also found that different efficacy of analgesia during delivery is not only related to the increased use of exogenous oxytocin during delivery but also related to the lactation initiation time, lactation volume and times of parturients. We suggest that the excellent effect of labor analgesia means that the higher block plane of epidural analgesia can inhibit the stimulation of the fetus to the birth canal and then reduce the release of oxytocin and prolactin, which results in prolonged lactation initiation time and increases the incidence of delayed onset of lactation. Studies have found that the mother’s breast feeding status within the first 24 h after vaginal delivery is related to the success rate of breastfeeding 6 months after vaginal delivery [10]. The lactation volume of healthy full-term newborns within 24 h after vaginal delivery is the average of 6 ml and the times of lactation is 10–12 times per day.Our study found that the lactation volume in Group M at 24, 48 and 72 h after vaginal delivery were more than those in Group E and Group P. Our study also found that the lactation volume in Group M at 48 and 72 h after vaginal delivery were more than those in Group E and Group P.This means the efficacy of epidural labor analgesia can affect the lactation volume during the whole 72 h and the moderate efficacy analgesia is of the most lactation volume among the three groups.This study found that there was no statistics difference of lactation times at 24 h after vaginal delivery among the three group .However, there was a statistics difference of lactation times among the three group at 48 and 72 h.It suggest that the efficacy of epidural labor analgesia does not affect the lactation times at the first 24 h.but can affect the lactation times at 48 and 72 h.And the lactation times of the moderate efficacy is of the most lactation times among the three groups.The above results maybe due to the prolongation of lactation initiation time in Group E and Group P.In addition, we also found that the lactation volume among the three groups increased accordingly at 24, 48 and 72 h after vaginal delivery, and that the lactation volume at 72 h was more than at 24 h and at 48 h and that at 48 h was more than 24 h, which suggested that the lactation volume gradually increased one day by one day among the three groups.

The LATCH assessment system was invented by JENSEN [19]. The assessment system is composed of 5 items, including hooking, swallowing sound, nipple type, comfort, and posture, with each item being scored 0, 1 and 2 points, respectively. The full score is 10 points. A total LATCH score of 8 to 10 points indicates that the breast feeding ability is good, 4–7 points indicate average ability, and 0–3 points indicate poor ability. Studies have reported that the higher the LATCH score is, the longer the expected breastfeeding time is, and parturients with a LATCH score ≥ 9 are 1.9 times more likely to breastfeed at 6 weeks postpartum than those with other scores [26]. It is extremely meaningful to evaluate the mother’s breast feeding situation in the early postpartum period, which is helpful for timely intervention and improvement of breastfeeding techniques [19]. Our study found that there was no statistically significant difference in the LATCH scores among the three groups. It is suggested that the efficacy of labor analgesia has no effect on the LATCH score, which reflects the overall situation of maternal breast feeding. This is related to the fact that the content of LATCH includes many factors of mother and newborn. However, there was a statistically significant difference in the LATCH score at 24, 48 and 72 h after vaginal delivery. We also found that the overall situation of breastfeeding among the three groups improved over time.

Our study has some limitations: (1) This study is a single-center study and The broad applicability of its findings may be limited. (2) This study is a retrospective study, and a strict randomized double-blind controlled study and should be performed to confirm the results of our study. (3) Since there are many factors affecting breastfeeding after vaginal delivery, the other confounding factors were adjusted into the three groups without significant differences, the results of our research should be adopted with caution.

## Conclusion

Our findings suggest that epidural labor analgesia with moderate analgesia efficacy have fewer cases of prolatin use, more lactation volume and time, shorter lactation initiation time, lower incidence of delayed onset of lactation and no effect on the LATCH score of breast feeding when compared with the excellent and poor efficacy of epidural labor analgesia.

### Supplementary Information

**Addtional file 1.**

## Data Availability

The datasets used and/or analyzed during the current study are available from the corresponding author on reasonable request.
